# Supplementary material for: Evaluating the technical efficiency and influencing factors of citrus fruits planting in China
Source: PLoS One. 2026 Apr 9;21(4):e0345871. doi: 10.1371/journal.pone.0345871 (PMC13065068; doi:10.1371/journal.pone.0345871)
Supplement: S1 Table — (DOCX) [file pone.0345871.s002.docx]

Output from the program FRONTIER (Version 4.1c)

instruction file = eg33-ins.txt

data file = eg33-dta.txt

Tech. Eff. Effects Frontier (see B&C 1993)

The model is a production function

The dependent variable is logged

the ols estimates are :

coefficient standard-error t-ratio

beta 0 -0.25626937E+02 0.10252819E+02 -0.24995016E+01

beta 1 0.40354334E+00 0.18472394E+00 0.21845753E+01

beta 2 -0.90904489E-02 0.23092018E-02 -0.39366196E+01

beta 3 0.13984864E+01 0.26092552E+01 0.53597148E+00

beta 4 0.58021131E+01 0.13848604E+01 0.41896736E+01

beta 5 0.18786395E+01 0.12358952E+01 0.15200638E+01

beta 6 -0.31276280E-01 0.29090842E-01 -0.10751246E+01

beta 7 0.21575488E-02 0.14815435E-01 0.14562845E+00

beta 8 -0.13638483E-01 0.82507679E-02 -0.16529956E+01

beta 9 -0.20692251E+00 0.16464643E+00 -0.12567689E+01

beta10 0.60684761E-02 0.16778511E+00 0.36168144E-01

beta11 -0.85629864E-01 0.58612754E-01 -0.14609425E+01

beta12 0.27624394E-01 0.21116167E+00 0.13082106E+00

beta13 -0.23253335E+00 0.83696359E-01 -0.27782971E+01

beta14 -0.11586431E+00 0.50739773E-01 -0.22835007E+01

sigma-squared 0.62495433E-01

log likelihood function = 0.52284478E+01

the estimates after the grid search were :

beta 0 -0.25491161E+02

beta 1 0.40354334E+00

beta 2 -0.90904489E-02

beta 3 0.13984864E+01

beta 4 0.58021131E+01

beta 5 0.18786395E+01

beta 6 -0.31276280E-01

beta 7 0.21575488E-02

beta 8 -0.13638483E-01

beta 9 -0.20692251E+00

beta10 0.60684761E-02

beta11 -0.85629864E-01

beta12 0.27624394E-01

beta13 -0.23253335E+00

beta14 -0.11586431E+00

delta 0 0.00000000E+00

delta 1 0.00000000E+00

delta 2 0.00000000E+00

delta 3 0.00000000E+00

delta 4 0.00000000E+00

delta 5 0.00000000E+00

delta 6 0.00000000E+00

sigma-squared 0.70629209E-01

gamma 0.41000000E+00

iteration = 0 func evals = 19 llf = 0.52980217E+01

-0.25491161E+02 0.40354334E+00-0.90904489E-02 0.13984864E+01 0.58021131E+01

0.18786395E+01-0.31276280E-01 0.21575488E-02-0.13638483E-01-0.20692251E+00

0.60684761E-02-0.85629864E-01 0.27624394E-01-0.23253335E+00-0.11586431E+00

0.00000000E+00 0.00000000E+00 0.00000000E+00 0.00000000E+00 0.00000000E+00

0.00000000E+00 0.00000000E+00 0.70629209E-01 0.41000000E+00

gradient step

iteration = 5 func evals = 44 llf = 0.53193721E+01

-0.25491079E+02 0.40349731E+00-0.88157897E-02 0.13987730E+01 0.58023783E+01

0.18788372E+01-0.31925488E-01 0.26108703E-02-0.14121415E-01-0.20703329E+00

0.62080636E-02-0.85070877E-01 0.27591704E-01-0.23276355E+00-0.11604274E+00

-0.62079427E-04 0.47943419E-03-0.80867367E-03-0.11721817E-02 0.56333815E-04

-0.58567828E-03 0.11222247E-03 0.70581619E-01 0.40999359E+00

iteration = 10 func evals = 65 llf = 0.57816551E+01

-0.25488417E+02 0.39135760E+00-0.88969802E-02 0.14077267E+01 0.58109251E+01

0.18829674E+01-0.29094423E-01 0.13966891E-02-0.13994473E-01-0.19984999E+00

-0.53520401E-02-0.82111185E-01 0.26966448E-01-0.23869575E+00-0.11101830E+00

0.39387789E-02 0.57730916E-01-0.68560791E-01-0.48405499E-02 0.60261403E-02

-0.41889911E-01 0.10252184E-01 0.69961033E-01 0.41423137E+00

iteration = 15 func evals = 82 llf = 0.67584131E+01

-0.25474572E+02 0.34152667E+00-0.83762435E-02 0.14539939E+01 0.58633069E+01

0.18830221E+01-0.26779707E-01 0.40569625E-02-0.12504165E-01-0.15861871E+00

-0.32449223E-01-0.89869825E-01 0.13775866E-01-0.26600106E+00-0.88587506E-01

0.16371111E-01 0.44472750E-01-0.37429313E+00 0.22473862E-01 0.27296859E-01

-0.71953457E-01 0.77285519E-01 0.72338298E-01 0.57782911E+00

iteration = 20 func evals = 101 llf = 0.99493294E+01

-0.25272157E+02 0.26984991E+00-0.84418036E-02 0.17788126E+01 0.62577073E+01

0.10020727E+01-0.30733160E-01 0.14108385E-01-0.68063580E-02-0.10001736E+00

0.51077422E-02-0.80218060E-01-0.41796716E-01-0.34324001E+00-0.40826822E-01

0.22265177E+00 0.57101346E-02-0.96616515E+00 0.27003243E-01 0.15382913E+00

-0.91075717E-01 0.12306789E+01 0.75140846E-01 0.76635395E+00

iteration = 25 func evals = 203 llf = 0.12114400E+02

-0.23939171E+02 0.32481060E+00-0.69522476E-02 0.21796357E+01 0.52330580E+01

0.11874523E+01-0.49285088E-01 0.25848888E-01-0.11822190E-01-0.51435239E-01

0.33656519E-01-0.12608239E+00-0.93378688E-01-0.28494984E+00-0.42060066E-01

0.79513197E+00-0.97217558E-01-0.10108885E+01-0.26221572E-01 0.28335676E+00

-0.17904778E-01 0.35542723E+01 0.72057514E-01 0.73911107E+00

iteration = 30 func evals = 319 llf = 0.13069768E+02

-0.12289329E+02 0.17496163E+00-0.51714340E-02 0.10335291E+01 0.41583466E+01

-0.11185257E+00-0.48529974E-01 0.40915678E-01-0.10993631E-01 0.25518145E-01

0.12321404E+00-0.81398665E-01-0.81308719E-01-0.27124960E+00-0.12480338E-01

0.38129375E+01-0.13267461E+00-0.89349920E+00-0.30898729E+00 0.10063203E+01

-0.77478645E-01 0.41849445E+01 0.77796755E-01 0.80833754E+00

iteration = 35 func evals = 444 llf = 0.15019845E+02

0.18071254E+00 0.13629889E-01-0.41451043E-02-0.21183930E+01 0.49096239E+01

-0.12536085E+01-0.31443608E-01 0.41993694E-01-0.86946524E-02-0.68127824E-01

0.26402192E+00-0.10422448E+00 0.11865866E+00-0.26687189E+00 0.10476442E-01

0.81548149E+01-0.21026422E+00-0.13194230E+01-0.72400645E+00 0.26130856E+01

-0.48638378E-01 0.41164314E+01 0.10306508E+00 0.83805293E+00

iteration = 40 func evals = 563 llf = 0.15965222E+02

-0.43547706E+01-0.20458231E-01-0.31227274E-02-0.76192167E+00 0.44836531E+01

-0.83014424E+00-0.33370948E-01 0.45758809E-01-0.74141425E-02-0.13398888E-01

0.22209023E+00-0.81945802E-01 0.16393235E-01-0.27423691E+00-0.17114898E-01

0.13819331E+02-0.26415785E+00-0.15416213E+01-0.13344105E+01 0.65569124E+01

0.10833489E+00 0.60006343E+01 0.14808101E+00 0.87013292E+00

iteration = 45 func evals = 676 llf = 0.16197174E+02

-0.42220523E+01-0.15218180E-01-0.31034965E-02-0.95700342E+00 0.46326273E+01

-0.81643509E+00-0.33304463E-01 0.45040439E-01-0.77077026E-02-0.33103313E-01

0.23249150E+00-0.89978834E-01 0.35273343E-01-0.27101001E+00-0.20383564E-01

0.16077807E+02-0.29825154E+00-0.18484749E+01-0.15679490E+01 0.83779658E+01

0.15055463E+00 0.62854290E+01 0.15124080E+00 0.85423764E+00

pt better than entering pt cannot be found

iteration = 47 func evals = 706 llf = 0.16228184E+02

-0.44535481E+01-0.15130776E-01-0.31302164E-02-0.91218525E+00 0.46332279E+01

-0.79286693E+00-0.33507630E-01 0.45158547E-01-0.74876579E-02-0.32497877E-01

0.23045602E+00-0.89465488E-01 0.32741578E-01-0.27157192E+00-0.21698204E-01

0.16306703E+02-0.30087095E+00-0.18960186E+01-0.15932435E+01 0.86501668E+01

0.15705788E+00 0.62934894E+01 0.15072243E+00 0.85132083E+00

the final mle estimates are :

coefficient standard-error t-ratio

beta 0 -0.44535481E+01 0.95379760E+01 -0.46692800E+00

beta 1 -0.15130776E-01 0.17166895E+00 -0.88139269E-01

beta 2 -0.31302164E-02 0.21346502E-02 -0.14663838E+01

beta 3 -0.91218525E+00 0.22642948E+01 -0.40285622E+00

beta 4 0.46332279E+01 0.11116075E+01 0.41680430E+01

beta 5 -0.79286693E+00 0.10879997E+01 -0.72873818E+00

beta 6 -0.33507630E-01 0.24981363E-01 -0.13413051E+01

beta 7 0.45158547E-01 0.13309822E-01 0.33928739E+01

beta 8 -0.74876579E-02 0.71489113E-02 -0.10473844E+01

beta 9 -0.32497877E-01 0.13111033E+00 -0.24786665E+00

beta10 0.23045602E+00 0.12526454E+00 0.18397547E+01

beta11 -0.89465488E-01 0.42429536E-01 -0.21085663E+01

beta12 0.32741578E-01 0.17666226E+00 0.18533431E+00

beta13 -0.27157192E+00 0.61649267E-01 -0.44051119E+01

beta14 -0.21698204E-01 0.49630634E-01 -0.43719376E+00

delta 0 0.16306703E+02 0.60117873E+01 0.27124550E+01

delta 1 -0.30087095E+00 0.12813396E+00 -0.23480970E+01

delta 2 -0.18960186E+01 0.89226409E+00 -0.21249522E+01

delta 3 -0.15932435E+01 0.63538970E+00 -0.25075060E+01

delta 4 0.86501668E+01 0.52851851E+01 0.16366819E+01

delta 5 0.15705788E+00 0.21570847E+00 0.72810255E+00

delta 6 0.62934894E+01 0.19340689E+01 0.32540150E+01

sigma-squared 0.15072243E+00 0.30635792E-01 0.49198150E+01

gamma 0.85132083E+00 0.64856301E-01 0.13126263E+02

log likelihood function = 0.16228184E+02

LR test of the one-sided error = 0.21999472E+02

with number of restrictions = 8

[note that this statistic has a mixed chi-square distribution]

number of iterations = 47

(maximum number of iterations set at : 100)

number of cross-sections = 7

number of time periods = 13

total number of observations = 91

thus there are: 0 obsns not in the panel

covariance matrix :

0.90972986E+02 -0.73302980E+00 0.38735423E-02 -0.18410934E+02 0.35098466E+00

-0.85055303E+01 0.74936620E-01 0.86106810E-02 0.11543081E-01 -0.24890216E+00

0.76421286E+00 -0.25011086E-01 0.10634079E+01 0.10985701E+00 0.28736844E+00

-0.35526345E+01 -0.27281355E+00 0.69017691E+00 0.71591627E+00 -0.13712531E+02

-0.55425608E+00 -0.52569986E+01 -0.20960401E-01 0.18628465E+00

-0.73302980E+00 0.29470228E-01 -0.14022527E-03 0.16310416E+00 -0.29830165E-01

0.59100991E-01 -0.35376379E-02 0.17440057E-03 -0.59149162E-03 0.40516921E-02

-0.72519186E-02 0.29575242E-03 -0.91200879E-02 -0.18903742E-03 -0.29749641E-03

-0.28911624E+00 0.34819902E-02 0.24799173E-01 0.27812181E-01 -0.13307611E+00

-0.54540683E-03 0.10139960E-01 -0.10017573E-02 -0.16006037E-02

0.38735423E-02 -0.14022527E-03 0.45567314E-05 0.20964423E-04 -0.75330181E-03

-0.41739465E-03 0.29572112E-05 0.90092020E-05 -0.22228281E-05 0.51878191E-04

0.90637393E-04 -0.15515876E-04 -0.56024819E-04 0.28002034E-04 -0.10751617E-04

0.25936757E-02 -0.60191581E-04 0.76691568E-04 -0.25766983E-03 0.70177355E-03

0.98216329E-04 0.11977457E-02 0.23154255E-04 0.41635347E-04

-0.18410934E+02 0.16310416E+00 0.20964423E-04 0.51270310E+01 -0.12998633E+01

0.13460555E+01 -0.27686123E-01 0.76604858E-02 -0.28461278E-02 0.16749376E+00

-0.12146734E+00 0.16251001E-01 -0.37088998E+00 -0.45917749E-02 -0.52539455E-01

0.51188443E+00 0.55736506E-01 0.75134590E-01 -0.12865802E+00 0.24410969E+01

0.14345957E+00 0.15894922E+01 0.61572827E-02 -0.25050802E-01

0.35098466E+00 -0.29830165E-01 -0.75330181E-03 -0.12998633E+01 0.12356713E+01

0.98861364E-01 0.12524289E-01 -0.82749553E-02 0.15915987E-02 -0.10673341E+00

-0.85582086E-02 -0.11853946E-01 0.13635980E+00 -0.24366477E-01 0.28590839E-02

0.69458477E-01 0.15676580E-01 -0.17464069E+00 -0.23221165E-02 0.19885934E+00

-0.49341204E-01 -0.53656756E+00 -0.29787585E-02 -0.91500817E-02

-0.85055303E+01 0.59100991E-01 -0.41739465E-03 0.13460555E+01 0.98861364E-01

0.11837434E+01 -0.23186262E-02 -0.35077542E-02 -0.18236389E-02 -0.54169410E-02

-0.10940853E+00 0.33258233E-02 -0.51054880E-01 -0.29851522E-02 -0.37336900E-01

0.69683265E+00 0.59157333E-02 -0.14420279E+00 -0.10239166E+00 0.15587831E+01

0.67299925E-01 0.44919704E+00 0.36622280E-02 -0.21513433E-01

0.74936620E-01 -0.35376379E-02 0.29572112E-05 -0.27686123E-01 0.12524289E-01

-0.23186262E-02 0.62406851E-03 -0.19737734E-03 0.70590600E-04 -0.16707861E-02

0.35293294E-03 -0.16306254E-04 0.22688769E-02 0.10241638E-03 -0.70024749E-04

0.26063949E-01 -0.29917380E-03 -0.26909046E-02 -0.23856583E-02 0.93821680E-02

-0.38170016E-03 -0.10870253E-01 0.15173793E-03 0.18207262E-03

0.86106810E-02 0.17440057E-03 0.90092020E-05 0.76604858E-02 -0.82749553E-02

-0.35077542E-02 -0.19737734E-03 0.17715136E-03 -0.21568544E-04 0.11094430E-02

0.39621594E-03 -0.33880807E-04 -0.10687927E-02 -0.83539609E-04 0.96231627E-04

0.39793817E-02 -0.88885401E-04 0.59558037E-04 -0.45689398E-03 0.28752628E-02

0.34462662E-03 0.84825809E-02 -0.23762957E-04 0.33746490E-04

0.11543081E-01 -0.59149162E-03 -0.22228281E-05 -0.28461278E-02 0.15915987E-02

-0.18236389E-02 0.70590600E-04 -0.21568544E-04 0.51106932E-04 -0.99069781E-04

0.94160552E-04 0.52849368E-04 0.17914116E-03 -0.70531209E-04 0.31759432E-04

0.75490655E-02 0.11196156E-04 -0.17059564E-02 -0.79236096E-03 0.82463031E-02

-0.41062014E-04 -0.19767490E-02 -0.53529044E-04 -0.12880897E-03

-0.24890216E+00 0.40516921E-02 0.51878191E-04 0.16749376E+00 -0.10673341E+00

-0.54169410E-02 -0.16707861E-02 0.11094430E-02 -0.99069781E-04 0.17189917E-01

-0.85698383E-03 0.18442674E-02 -0.18083792E-01 -0.28922171E-02 -0.34271742E-04

-0.86604333E-01 0.25935784E-02 0.22973764E-01 0.72313861E-02 -0.41479707E-01

0.39766386E-02 0.49546775E-01 -0.28759405E-03 0.49867819E-03

0.76421286E+00 -0.72519186E-02 0.90637393E-04 -0.12146734E+00 -0.85582086E-02

-0.10940853E+00 0.35293294E-03 0.39621594E-03 0.94160552E-04 -0.85698383E-03

0.15691204E-01 -0.26401614E-02 0.33333297E-02 0.18161662E-02 0.12390925E-02

0.68629965E-01 -0.31139473E-02 -0.69811615E-03 -0.47469285E-02 -0.48940641E-01

0.64311979E-03 -0.11278842E-01 0.31544307E-03 0.17371409E-02

-0.25011086E-01 0.29575242E-03 -0.15515876E-04 0.16251001E-01 -0.11853946E-01

0.33258233E-02 -0.16306254E-04 -0.33880807E-04 0.52849368E-04 0.18442674E-02

-0.26401614E-02 0.18002655E-02 -0.10776233E-02 -0.77744165E-03 0.26894877E-03

-0.13647951E-01 0.53007444E-03 0.38123941E-02 0.12446988E-02 -0.12308731E-03

-0.91053399E-03 -0.84926398E-02 -0.12591152E-03 -0.44101781E-04

0.10634079E+01 -0.91200879E-02 -0.56024819E-04 -0.37088998E+00 0.13635980E+00

-0.51054880E-01 0.22688769E-02 -0.10687927E-02 0.17914116E-03 -0.18083792E-01

0.33333297E-02 -0.10776233E-02 0.31209553E-01 0.76336343E-03 0.31229094E-02

-0.27557287E-01 -0.37763445E-02 -0.14778344E-01 0.78053695E-02 -0.13149198E+00

-0.11413770E-01 -0.12136892E+00 -0.44694063E-03 0.79770521E-03

0.10985701E+00 -0.18903742E-03 0.28002034E-04 -0.45917749E-02 -0.24366477E-01

-0.29851522E-02 0.10241638E-03 -0.83539609E-04 -0.70531209E-04 -0.28922171E-02

0.18161662E-02 -0.77744165E-03 0.76336343E-03 0.38006322E-02 -0.38061082E-03

0.50009091E-01 -0.27581884E-02 -0.14310533E-02 -0.45315850E-02 0.11053791E-01

0.16806444E-02 0.11411343E-01 0.45073001E-03 0.36530217E-03

0.28736844E+00 -0.29749641E-03 -0.10751617E-04 -0.52539455E-01 0.28590839E-02

-0.37336900E-01 -0.70024749E-04 0.96231627E-04 0.31759432E-04 -0.34271742E-04

0.12390925E-02 0.26894877E-03 0.31229094E-02 -0.38061082E-03 0.24631999E-02

-0.11104729E+00 0.11110115E-02 0.12740770E-01 0.12866231E-01 -0.12074184E+00

-0.60783076E-02 -0.27499030E-01 -0.44598367E-03 0.94633221E-03

-0.35526345E+01 -0.28911624E+00 0.25936757E-02 0.51188443E+00 0.69458477E-01

0.69683265E+00 0.26063949E-01 0.39793817E-02 0.75490655E-02 -0.86604333E-01

0.68629965E-01 -0.13647951E-01 -0.27557287E-01 0.50009091E-01 -0.11104729E+00

0.36141587E+02 -0.50490162E+00 -0.43615856E+01 -0.38096946E+01 0.29458138E+02

0.90884180E+00 0.60411738E+01 0.92200299E-01 -0.97848766E-01

-0.27281355E+00 0.34819902E-02 -0.60191581E-04 0.55736506E-01 0.15676580E-01

0.59157333E-02 -0.29917380E-03 -0.88885401E-04 0.11196156E-04 0.25935784E-02

-0.31139473E-02 0.53007444E-03 -0.37763445E-02 -0.27581884E-02 0.11110115E-02

-0.50490162E+00 0.16418310E-01 0.69871269E-01 0.50634280E-01 -0.37903926E+00

-0.13991395E-01 -0.71090466E-01 -0.12132283E-02 0.12036474E-02

0.69017691E+00 0.24799173E-01 0.76691568E-04 0.75134590E-01 -0.17464069E+00

-0.14420279E+00 -0.26909046E-02 0.59558037E-04 -0.17059564E-02 0.22973764E-01

-0.69811615E-03 0.38123941E-02 -0.14778344E-01 -0.14310533E-02 0.12740770E-01

-0.43615856E+01 0.69871269E-01 0.79613521E+00 0.45909222E+00 -0.40159633E+01

-0.10636390E+00 -0.33324997E+00 -0.39334868E-02 0.26058770E-01

0.71591627E+00 0.27812181E-01 -0.25766983E-03 -0.12865802E+00 -0.23221165E-02

-0.10239166E+00 -0.23856583E-02 -0.45689398E-03 -0.79236096E-03 0.72313861E-02

-0.47469285E-02 0.12446988E-02 0.78053695E-02 -0.45315850E-02 0.12866231E-01

-0.38096946E+01 0.50634280E-01 0.45909222E+00 0.40372006E+00 -0.31694708E+01

-0.99275991E-01 -0.65815883E+00 -0.97907972E-02 0.11009230E-01

-0.13712531E+02 -0.13307611E+00 0.70177355E-03 0.24410969E+01 0.19885934E+00

0.15587831E+01 0.93821680E-02 0.28752628E-02 0.82463031E-02 -0.41479707E-01

-0.48940641E-01 -0.12308731E-03 -0.13149198E+00 0.11053791E-01 -0.12074184E+00

0.29458138E+02 -0.37903926E+00 -0.40159633E+01 -0.31694708E+01 0.27933181E+02

0.79515350E+00 0.47022919E+01 0.56358873E-01 -0.13784412E+00

-0.55425608E+00 -0.54540683E-03 0.98216329E-04 0.14345957E+00 -0.49341204E-01

0.67299925E-01 -0.38170016E-03 0.34462662E-03 -0.41062014E-04 0.39766386E-02

0.64311979E-03 -0.91053399E-03 -0.11413770E-01 0.16806444E-02 -0.60783076E-02

0.90884180E+00 -0.13991395E-01 -0.10636390E+00 -0.99275991E-01 0.79515350E+00

0.46530142E-01 0.16802934E+00 0.30333107E-02 -0.26802359E-02

-0.52569986E+01 0.10139960E-01 0.11977457E-02 0.15894922E+01 -0.53656756E+00

0.44919704E+00 -0.10870253E-01 0.84825809E-02 -0.19767490E-02 0.49546775E-01

-0.11278842E-01 -0.84926398E-02 -0.12136892E+00 0.11411343E-01 -0.27499030E-01

0.60411738E+01 -0.71090466E-01 -0.33324997E+00 -0.65815883E+00 0.47022919E+01

0.16802934E+00 0.37406227E+01 0.34438661E-01 0.71865893E-02

-0.20960401E-01 -0.10017573E-02 0.23154255E-04 0.61572827E-02 -0.29787585E-02

0.36622280E-02 0.15173793E-03 -0.23762957E-04 -0.53529044E-04 -0.28759405E-03

0.31544307E-03 -0.12591152E-03 -0.44694063E-03 0.45073001E-03 -0.44598367E-03

0.92200299E-01 -0.12132283E-02 -0.39334868E-02 -0.97907972E-02 0.56358873E-01

0.30333107E-02 0.34438661E-01 0.93855173E-03 0.91933122E-03

0.18628465E+00 -0.16006037E-02 0.41635347E-04 -0.25050802E-01 -0.91500817E-02

-0.21513433E-01 0.18207262E-03 0.33746490E-04 -0.12880897E-03 0.49867819E-03

0.17371409E-02 -0.44101781E-04 0.79770521E-03 0.36530217E-03 0.94633221E-03

-0.97848766E-01 0.12036474E-02 0.26058770E-01 0.11009230E-01 -0.13784412E+00

-0.26802359E-02 0.71865893E-02 0.91933122E-03 0.42063398E-02

technical efficiency estimates :

firm year eff.-est.

1 1 0.92986893E+00

2 1 0.93289434E+00

3 1 0.53943939E+00

4 1 0.40193892E+00

5 1 0.25639144E+00

6 1 0.76861159E+00

7 1 0.61362796E+00

1 2 0.88209807E+00

2 2 0.92795271E+00

3 2 0.51471150E+00

4 2 0.36741795E+00

5 2 0.33854764E+00

6 2 0.84284428E+00

7 2 0.72837685E+00

1 3 0.94773370E+00

2 3 0.93773853E+00

3 3 0.89808459E+00

4 3 0.71248777E+00

5 3 0.80379962E+00

6 3 0.89031641E+00

7 3 0.87001333E+00

1 4 0.95226814E+00

2 4 0.86950708E+00

3 4 0.62346794E+00

4 4 0.89331240E+00

5 4 0.73799151E+00

6 4 0.92316198E+00

7 4 0.88467107E+00

1 5 0.94846269E+00

2 5 0.95916599E+00

3 5 0.59551374E+00

4 5 0.82418832E+00

5 5 0.72244699E+00

6 5 0.95027829E+00

7 5 0.65732963E+00

1 6 0.94630268E+00

2 6 0.93713930E+00

3 6 0.74167629E+00

4 6 0.69744502E+00

5 6 0.83577629E+00

6 6 0.96420307E+00

7 6 0.79573382E+00

1 7 0.96426756E+00

2 7 0.96475098E+00

3 7 0.87137764E+00

4 7 0.76703636E+00

5 7 0.94541971E+00

6 7 0.93458369E+00

7 7 0.73063431E+00

1 8 0.93588218E+00

2 8 0.96237392E+00

3 8 0.89223381E+00

4 8 0.89871253E+00

5 8 0.90981743E+00

6 8 0.93475062E+00

7 8 0.86486388E+00

1 9 0.94047999E+00

2 9 0.95912304E+00

3 9 0.84050260E+00

4 9 0.81124086E+00

5 9 0.90623561E+00

6 9 0.93524475E+00

7 9 0.77781557E+00

1 10 0.95904111E+00

2 10 0.97063627E+00

3 10 0.88980192E+00

4 10 0.92008771E+00

5 10 0.62191373E+00

6 10 0.95548272E+00

7 10 0.72182062E+00

1 11 0.93693838E+00

2 11 0.95838648E+00

3 11 0.89815826E+00

4 11 0.62791485E+00

5 11 0.92409321E+00

6 11 0.91665509E+00

7 11 0.72573551E+00

1 12 0.89337776E+00

2 12 0.94339964E+00

3 12 0.84870759E+00

4 12 0.93357474E+00

5 12 0.94014161E+00

6 12 0.94415154E+00

7 12 0.88988175E+00

1 13 0.95555318E+00

2 13 0.97159088E+00

3 13 0.95038490E+00

4 13 0.92323893E+00

5 13 0.77214425E+00

6 13 0.94675386E+00

7 13 0.95464175E+00

mean efficiency = 0.83743399E+00

summary of panel of observations:

(1 = observed, 0 = not observed)

t: 1 2 3 4 5 6 7 8 9 10 11 12 13

n

1 1 1 1 1 1 1 1 1 1 1 1 1 1 13

2 1 1 1 1 1 1 1 1 1 1 1 1 1 13

3 1 1 1 1 1 1 1 1 1 1 1 1 1 13

4 1 1 1 1 1 1 1 1 1 1 1 1 1 13

5 1 1 1 1 1 1 1 1 1 1 1 1 1 13

6 1 1 1 1 1 1 1 1 1 1 1 1 1 13

7 1 1 1 1 1 1 1 1 1 1 1 1 1 13

7 7 7 7 7 7 7 7 7 7 7 7 7 91
