# Supplementary material for: Evaluating the technical efficiency and influencing factors of citrus fruits planting in China
Source: PLoS One. 2026 Apr 9;21(4):e0345871. doi: 10.1371/journal.pone.0345871 (PMC13065068; doi:10.1371/journal.pone.0345871)
Supplement: Attachment — Submitted filename: Review of Technical Efficiency Citrus .docx [file pone.0345871.s004.docx]

**Review of "Evaluating the Technical Efficiency and Influencing Factors of Citrus Fruits Planting in China"**

This paper presents an interesting study on the technical efficiency of citrus fruit planting in China. The authors employ a stochastic frontier analysis (SFA) model with a beyond logarithmic production function to analyze panel data from seven major citrus-producing provinces and cities in China from 2012 to 2022. The topic is relevant, particularly given the importance of the citrus industry in China and the need to improve its production efficiency. The methodology is generally sound, and the results provide some useful insights. However, there are some weaknesses that need to be addressed before the manuscript can be considered for publication.

**Weaknesses:**

- **Justification of Variables:** While the authors explain the variables used in the model, a more in-depth justification for their inclusion and potential impact would be beneficial. For example, the choice of "indirect material and service input" could be further elaborated.
- **Discussion of Limitations:** The authors should include a more thorough discussion of the limitations of their study. This could include potential biases in the data, limitations of the model, and the generalizability of the results.

**Specific Comments and Suggestions for Revision:**

1. **Introduction:**
   - The introduction provides a good overview of the citrus industry in China. However, it could benefit from a clearer statement of the research gap and the specific contributions of this study.
2. **Literature Review:**
   - The literature review provides a good overview of previous studies on efficiency analysis in agriculture, particularly in the citrus sector. However, it could be improved by:
     - Explicitly stating how this study builds upon or differs from previous research.
3. **Model Construction:**
   - The model construction section is generally well-explained. However:
     - The justification for including specific variables in the inefficiency function could be strengthened.
4. **Results and Discussion:**
   - The results are presented clearly, and the discussion is generally good. However:
     - The discussion could be more in-depth, providing more context for the findings and comparing them with previous research.
     - The authors should discuss the policy implications of their findings in more detail.
5. **Conclusions:**
   - The conclusions summarize the main findings of the study. However:
     - The authors should reiterate the limitations of the study and suggest directions for future research.
