# Supplementary material for: Evaluating the technical efficiency and influencing factors of citrus fruits planting in China
Source: PLoS One. 2026 Apr 9;21(4):e0345871. doi: 10.1371/journal.pone.0345871 (PMC13065068; doi:10.1371/journal.pone.0345871)
Supplement: Attachment — Submitted filename: Response to Reviewers.docx [file pone.0345871.s006.docx]

**Review of "Evaluating the Technical Efficiency and Influencing Factors of Citrus Fruits Planting in China"**

This paper presents an interesting study on the technical efficiency of citrus fruit planting in China. The authors employ a stochastic frontier analysis (SFA) model with a beyond logarithmic production function to analyze panel data from seven major citrus-producing provinces and cities in China from 2012 to 2022. The topic is relevant, particularly given the importance of the citrus industry in China and the need to improve its production efficiency. The methodology is generally sound, and the results provide some useful insights. However, there are some weaknesses that need to be addressed before the manuscript can be considered for publication.

**Weaknesses:**

- **Justification of Variables:** While the authors explain the variables used in the model, a more in-depth justification for their inclusion and potential impact would be beneficial. For example, the choice of "indirect material and service input" could be further elaborated.
- The rationale for the selection of these variables was mainly based on the actual situation of citrus planting, the combination of previous relevant studies, and the availability of data, which has been described in the text.
- **Discussion of Limitations:** The authors should include a more thorough discussion of the limitations of their study. This could include potential biases in the data, limitations of the model, and the generalizability of the results.
- The limitations of the study are explained. See the “Discussion” section for details.

**Specific Comments and Suggestions for Revision:**

1. **Introduction:**
   - The introduction provides a good overview of the citrus industry in China. However, it could benefit from a clearer statement of the research gap and the specific contributions of this study.
   - Changes have been made, as requested, to add the specific contributions of the study, as detailed at the end of the first paragraph of the Introduction.
2. **Literature Review:**
   - The literature review provides a good overview of previous studies on efficiency analysis in agriculture, particularly in the citrus sector. However, it could be improved by:
     - Explicitly stating how this study builds upon or differs from previous research.
     - Changes have been made, as requested, to add the specific contributions of the study, as detailed at the end of the first paragraph of the Introduction.
3. **Model Construction:**
   - The model construction section is generally well-explained. However:
     - The justification for including specific variables in the inefficiency function could be strengthened.
     - The specific variables included in the inefficiency function are based on the actual production situation, previous studies, and data availability, as described in the “Model Construction” section.
4. **Results and Discussion:**
   - The results are presented clearly, and the discussion is generally good. However:
     - The discussion could be more in-depth, providing more context for the findings and comparing them with previous research.
     - The authors should discuss the policy implications of their findings in more detail.
     - Comparisons with previous studies are made and possible policy implications of the findings are described in the Discussion section.
5. **Conclusions:**
   - The conclusions summarize the main findings of the study. However:
     - The authors should reiterate the limitations of the study and suggest directions for future research.
     - The limitations of the study are explained and future research directions are proposed, as detailed in the Discussion section.

Reviewer #2: -The manuscript should be structured to include the following sections: an Introduction, which provides background information and outlines the study's objectives; Materials and Methods, detailing the experimental design, data collection, and analytical techniques; Results, presenting the key findings objectively; and Discussion, interpreting the results in the context of existing literature and highlighting their implications.

The manuscript has been revised according to the structure of “Introduction, Materials and Methods, Results, Discussion”.

-The manuscript requires revision to improve the quality of the English language."

-Key words: Aren’t keywords usually supposed to be different from words already mentioned in title and abstract?

Keywords have been deleted.

-The introduction lacks appropriate references to support key statements and provide context for the study.

The structure of the article has been restructured, and there is a review and introduction of relevant research in the context of the study.

-Why has the author separated the Introduction and Literature Review into distinct sections?

The article structure was restructured, and the introduction and article review were merged.

-The line spacing format is inconsistent.

Adjusted to harmonized spacing.

-The citation format in the text is inconsistent, as the author alternates between using numbered references and author names for citations.

-Additionally, the citation style should include only the first author’s name, followed by 'et al.' when citing multiple authors.

The citation format of the references has been revised as required.

-What does the author mean by the term “natural disasters in agriculture”?

Natural disasters in agriculture mainly refer to floods, droughts, etc.

-Huanglongbing (HLB) is an important factor affecting citrus production efficiency in China, and its inclusion in the study will significantly enhance this finding. It is highly recommended to include this factor in the study. Also, the study does not sufficiently explore sustainable alternatives such as organic farming, integrated pest management (IPM), or biofertilizers on citrus production in China.

-The author did not provide sufficient justification or supporting evidence for conducting this research.

-The conclusion is overly lengthy and could be more concise.

Huanglongbing (HLB) is an important factor affecting citrus production efficiency in China。However, it was not included in the analysis of influencing factors in this paper due to some difficulties in data collection and acquisition, which could be included in subsequent studies.

In addition, the structure and content of the article has been reorganized, splitting the original conclusion into two parts, conclusion and discussion, which makes a bit more sense.
