# Supplementary material for: Evaluating the technical efficiency and influencing factors of citrus fruits planting in China
Source: PLoS One. 2026 Apr 9;21(4):e0345871. doi: 10.1371/journal.pone.0345871 (PMC13065068; doi:10.1371/journal.pone.0345871)
Supplement: Attachment — Submitted filename: Response_to_Reviewers_auresp_2.docx [file pone.0345871.s007.docx]

Reviewer #2: I have a few suggestions and comments to improve the clarity, accuracy, and structure of the manuscript:

Introduction:
-The study is being submitted in 2025, yet the Introduction heavily relies on data from 2022. Please consider updating your citrus production statistics (both globally and for China) to reflect the most recent data available.

The data has been updated to the latest version as requested. Please refer to the introduction section for details.

-The Introduction would benefit from additional, more recent references, particularly when discussing global and national citrus production trends and the importance of citrus in China's agricultural economy.
-The current Introduction dives directly into citrus importance and efficiency measurements without first framing the broader challenges facing citrus production (e.g., climate change, pest pressure, resource inefficiency, market access). I suggest starting with a short paragraph that clearly outlines the general problems in citrus production, and then narrowing the focus to technical efficiency, highlighting previous studies and where the research gap lies.

The introduction section has been appropriately edited to include common issues faced in citrus planting.

-Please ensure that the in-text citation format is consistent throughout the manuscript. Currently, the text switches between numbered references and author-date narrative formats (e.g., "Xiong Wei et al." vs. [8]). Align with the journal’s required citation style.

The revisions have been made as requested. The citation format is consistent with that of the journal's reference list.

-In the section where the objectives of the study are stated, please clearly mention the seven provinces/cities included in your panel data analysis. This will help readers immediately understand the geographical scope and relevance of the study.

Seven major citrus growing regions were added at the end of the introduction.

Material and methods
Many parts of the material and methods need to be moved to the introduction section.
For example:
“Citrus planting is a typical labor-intensive task [22] , and from a practical point of view, citrus harvests are affected by labor intensity, fertilizer and pesticide inputs, etc., based on the current situation and the caliber of agricultural statistics and data availability…”.
“The production activities of citrus planting are profoundly affected by the impacts of the natural and social environments. For example, climate has an important impact on citrus planting[24]. Citrus frost damage not only affects plant production but also has a sustained impact on production over a number of years, and the problem of transport accessibility in citrus-producing areas has a direct impact on whether citrus crops can be marketed 6 successfully[25].”

This section mainly aims to objectively describe factors that may affect the inefficiency of citrus production, thereby incorporating regional economic differences, climatic differences, transportation infrastructure conditions, etc. into the analytical framework to construct an inefficiency function.

The data source
The first paragraph of data sources should be moved to the introduction for objective goals.
This section provides a brief overview of why these seven regions were selected as samples for studying citrus efficiency issues.

The model estimation and analysis
This section presents the core findings of your model estimation and variable significance testing. I strongly recommend incorporating this section into the Results section. This would improve the organization of your manuscript and help the reader follow your analytical progression.
Discussion
The Discussion section requires stronger connections with your model results, more nuanced interpretation, and clearer implications for practice and policy. I encourage you to deepen the analysis and focus on evidence-based recommendations. These improvements will significantly enhance the scientific contribution of your work.

Modifications have been made as requested.

-The English language and writing quality need improvement to enhance clarity and academic readability.

Reviewer #3: Abstract: Ok. The last three paragraphs of the summary are not significant to the text.

Introduction: Use more up-to-date statistics. In 2023, global citrus production was 217 million tons. Data from FAOSTAT <https://www.fao.org/faostat/es/#data/QCL> Also, data from China, reporting 95 million tons in 2023. Update performance data, in 2023 it was 14,800 kg/hm2, compared to Paraguay, Indonesia or South Africa which is three times higher. There are no DEA studies that link production with environmental or soil aspects? Are there any studies on the productivity of countries with high citrus yields?
The relevant data has been updated in accordance with the opinion.

Materials and methods: Social data can be very subjective, so how do you handle qualitative data? Also, when using previous studies, they should be from locations like the one in this study. It is suggested that a location map of the study area be added. When using scales in plot size, indicate that it represents large or small scales in a unit of measurement. During the analysis period, associate data with regional climate phenomena.

Based on actual production conditions, combined with previous relevant research and data availability, regional economic differences, climate differences, transportation infrastructure conditions, and other factors are incorporated into the analytical framework to objectively describe factors that may affect the inefficiency of citrus production and construct an inefficiency function.

Results: There is information found in materials and methods that could be reported in results. Adding a graph or map by region or zone would be helpful for comparing information.

The relevant results obtained in the article have been summarized in the conclusion section.

Discussion: The discussion could be supported by a graph to reinforce what is stated in the text. A basic analysis of the information is conducted; the data used in the models could help foster further discussion, given the time and space.

No conclusions to the work?

Figures have been added to the text to clearly illustrate the basic situation of citrus production value in the main citrus-producing regions. The conclusion section has been expanded.
