# Supplementary material for: Evaluating the technical efficiency and influencing factors of citrus fruits planting in China
Source: PLoS One. 2026 Apr 9;21(4):e0345871. doi: 10.1371/journal.pone.0345871 (PMC13065068; doi:10.1371/journal.pone.0345871)
Supplement: Attachment — Submitted filename: Response_to_Reviewers_auresp_3.docx [file pone.0345871.s008.docx]

Reviewers' comments:

Reviewer's Responses to Questions

**Comments to the Author**

1. If the authors have adequately addressed your comments raised in a previous round of review and you feel that this manuscript is now acceptable for publication, you may indicate that here to bypass the “Comments to the Author” section, enter your conflict of interest statement in the “Confidential to Editor” section, and submit your "Accept" recommendation.

Reviewer #2: (No Response)

Reviewer #3: All comments have been addressed

2. Is the manuscript technically sound, and do the data support the conclusions?

The manuscript must describe a technically sound piece of scientific research with data that supports the conclusions. Experiments must have been conducted rigorously, with appropriate controls, replication, and sample sizes. The conclusions must be drawn appropriately based on the data presented.

Reviewer #2: Yes

Reviewer #3: Yes

3. Has the statistical analysis been performed appropriately and rigorously?

Reviewer #2: I Don't Know

Reviewer #3: Yes

Statistical analysis has been rigorously implemented.

4. Have the authors made all data underlying the findings in their manuscript fully available?

The [PLOS Data policy](http://track.editorialmanager.com/CL0/http:%2F%2Fwww.plosone.org%2Fstatic%2Fpolicies.action%23sharing/1/010f019a3013f3e1-f31b9294-b852-4270-9890-2eef11df5a11-000000/8gRW3ah24gx65iiC5HaV14WX1nUhBm53MvMQYqITTuQ=235) requires authors to make all data underlying the findings described in their manuscript fully available without restriction, with rare exception (please refer to the Data Availability Statement in the manuscript PDF file). The data should be provided as part of the manuscript or its supporting information, or deposited to a public repository. For example, in addition to summary statistics, the data points behind means, medians and variance measures should be available. If there are restrictions on publicly sharing data—e.g. participant privacy or use of data from a third party—those must be specified.

Reviewer #2: Yes

Reviewer #3: Yes

5. Is the manuscript presented in an intelligible fashion and written in standard English?

PLOS ONE does not copyedit accepted manuscripts, so the language in submitted articles must be clear, correct, and unambiguous. Any typographical or grammatical errors should be corrected at revision, so please note any specific errors here.

Reviewer #2: No

Reviewer #3: Yes

The language throughout the document has been refined. For details, please refer to the revised version.

6. Review Comments to the Author

Please use the space provided to explain your answers to the questions above. You may also include additional comments for the author, including concerns about dual publication, research ethics, or publication ethics. (Please upload your review as an attachment if it exceeds 20,000 characters)

Reviewer #2: Reviewer Comment:
The manuscript has been notably improved; however, the English language still requires careful revision to enhance readability and clarity. The conclusion should be moved to the end of the manuscript to follow standard structure. Additionally, the quality and presentation of the tables should be improved.

Reviewer #3: A note: the conclusion is the final point of the manuscript. In the text, the conclusion was placed first and the discussion second. Change the order for better understanding.

Thank you for the reviewers' comments. The language throughout the manuscript has been further revised. The conclusion section has been moved to the end of the paper. The formatting of the tables in the manuscript has been adjusted，please refer to the revised version for details.

7. PLOS authors have the option to publish the peer review history of their article ([what does this mean?](https://track.editorialmanager.com/CL0/https:%2F%2Fjournals.plos.org%2Fplosone%2Fs%2Feditorial-and-peer-review-process%23loc-peer-review-history/1/010f019a3013f3e1-f31b9294-b852-4270-9890-2eef11df5a11-000000/wSMeakhFToKxtU1h6pBzaaypPxlT0HEio07LI3CSTSw=235)). If published, this will include your full peer review and any attached files.

If you choose “no”, your identity will remain anonymous but your review may still be made public.

**Do you want your identity to be public for this peer review?** For information about this choice, including consent withdrawal, please see our [Privacy Policy](https://track.editorialmanager.com/CL0/https:%2F%2Fwww.plos.org%2Fprivacy-policy/1/010f019a3013f3e1-f31b9294-b852-4270-9890-2eef11df5a11-000000/RFN18k4IcenMjUPAbfsJtv_4qzIdvfeC1foC--cAmVM=235).

Reviewer #2: **Yes:** Saoussen Ben Abdallah

Reviewer #3: **Yes:** LUIS ALBERTO OLVERA VARGAS
