# Supplementary material for: Evaluating the technical efficiency and influencing factors of citrus fruits planting in China
Source: PLoS One. 2026 Apr 9;21(4):e0345871. doi: 10.1371/journal.pone.0345871 (PMC13065068; doi:10.1371/journal.pone.0345871)
Supplement: Attachment — Submitted filename: Response_to_Reviewers_auresp_4.docx [file pone.0345871.s009.docx]

Reviewers' comments:

Reviewer #2: Introduction

In the section beginning with “Citrus, indigenous to China … until the competitiveness of the industry”, no reference is provided to support the statements made. Appropriate citations should be added to substantiate this information.

Additionally, the authors report data up to 2023; however, given that we are now in 2025, it would strengthen the manuscript to include the most recent available data, along with corresponding references, to provide an updated perspective.

Appropriate citations have been added as requested. All data throughout the text has been updated to 2024 (the 2025 yearbook contains 2024 data), including cultivation data for the seven sample provinces and municipalities discussed later. Re-calculations and analyses have been performed based on the latest data.

Regarding the citation of Chen Xinjian et al., the reference number for this study should be clearly indicated in the text to ensure consistency with the reference list.

The revisions have been made as requested.

It is recommended that the authors avoid using the phrase “this paper” and instead use “this study” or “this research”.

The revisions have been made as requested.

Material and methods

It would improve the clarity and logical flow of this section if the authors first describe the data sources, followed by the model construction used in the study.

Given the specific metrics involved in the model, the subsequent data has been organized according to these metrics to facilitate better understanding and correspondence.

Results

After the Materials and Methods section, the subheading Results is missing and should be added.

The results section has been added; see the main text for details.

For Table 1, How the standard deviation of Indirect material and service input per mu (yuan) (279.24) exceeds the corresponding mean value (267.02)? The authors should explain this variability or verify the accuracy of the reported data.

A standard deviation greater than the mean indicates a high degree of data dispersion, the presence of outliers, and a skewed distribution. This has been appropriately explained in the relevant section.

In addition, no statistical analyses are reported for Table 1, Table 3, Figure 2, and Figure 3. Interpretation or discussion of differences and significance should only be presented if appropriate statistical tests were conducted. The authors are encouraged to include the relevant statistical analyses.

The relevant sections have been revised accordingly.

Discussion

The Discussion section needs to be improved. The authors should provide a deeper interpretation of their results, discussing it with previous studies. In particular, the findings could be better contextualized by comparing them with similar studies conducted in other countries, such as Brazil, to highlight similarities, differences, and broader implications.

In addition, the authors are encouraged to engage with more recent literature to strengthen the discussion. For example, recently published studies (e.g., Bin Fan et al., 2025) could be used to support, contrast, or expand upon the current findings and demonstrate the study’s importance.

Appropriate improvements have been made as required.

Conclusion

The Conclusion section is excessively long, and several parts contain interpretative content that would be more appropriately placed in the Discussion section. The authors are encouraged to streamline the conclusion by focusing on the key findings and main take home messages, while moving detailed explanations and comparative discussion to the Discussion section.

Appropriate edits have been made to the content of the conclusion section.
